# Correction: RecA filament sliding on DNA facilitates homology search

**DOI:** 10.7554/eLife.00488

**Published:** 2013-02-20

**Authors:** Kaushik Ragunathan, Cheng Liu, Taekjip Ha

**Keywords:** Single Molecule, FRET, DNA repair, Homologous Recombination, E. coli

Ragunathan K, Liu C, Ha T. 2012. RecA filament sliding on DNA facilitates homology search. *eLife* **1**:e00067. doi: http://dx.doi.org/10.7554/eLife.00067. Published 13 December 2012

The author affiliations for Kaushik Ragunathan and Taekjip Ha were incorrect. The correct author affiliations are:

Kaushik Ragunathan^1,2,3†a^, Cheng Liu^4†b^, Taekjip Ha^1,2,3,4,5*^

^1^Center for Biophysics and Computational Biology, University of Illinois at Urbana-Champaign, Urbana, United States

^2^Center for the Physics of Living Cells, University of Illinois at Urbana-Champaign, Urbana, United States

^3^Institute for Genomic Biology, University of Illinois at Urbana-Champaign, Urbana, United States

^4^Department of Physics, University of Illinois at Urbana-Champaign, Urbana, United States

^5^Howard Hughes Medical Institute, University of Illinois at Urbana-Champaign, Urbana, United States

Present address: ^†a^Department of Cell Biology, Harvard Medical School, Boston, United States; ^†b^Center for Research Computing, University of Notre Dame, Notre Dame, United States

The article has been corrected accordingly.

