# Supplementary material for: RecA filament sliding on DNA facilitates homology search
Source: eLife. 2012 Dec 13;1:e00067. doi: 10.7554/eLife.00067 (PMC3510455; doi:10.7554/eLife.00067)
Supplement: Supplementary file 1. — DNA sequences used in measurements DOI: http://dx.doi.org/10.7554/eLife.00067.017 [file elife00067s001.pdf]

Materials and Methods-Source Data 1. DNA sequences used in measurements

Name	DNA sequence (5' -> 3')	Experiment
biotin_DNA	/Biotin/TGG CGA CGG CAG CGA GGC/Cy5/	For template ssDNA immobilization
Nh21+_template	CTT TTC ATC ACG TTG TTA GAT GCC TCG CTG CCG TCG CCA	Non-homologous ssDNA sequence, 21nt
Nh39+_template	TTT ACT TGT ACT TCA TTC ATT CAC ATT CCT ATC ATG TTT GCC TCG CTG CCG TCG CCA	Non-homologous ssDNA sequence, 39nt
T30 Nh39+_template	T30 TTT ACT TGT ACT TCA TTC ATT CAC ATT CCT ATC ATG TTT GCC TCG CTG CCG TCG CCA	Non-homologous ss DNA sequence, 69nt
T60 Nh39+_template	T60 TTT ACT TGT ACT TCA TTC ATT CAC ATT CCT ATC ATG TTT GCC TCG CTG CCG TCG CCA	Non-homologous ssDNA sequence, 99nt
99nt_3color_template	T60 TTT AC/Cy7-dT/ TGT ACT TCA TTC ATT CAC ATT CCT ATC ATG TTT GCC TCG CTG CCG TCG CCA	Template for 3-color experiments
poly T50	T50 GCC TCG CTG CCG TCG CCA	poly T DNA
2X 6bp pdT	TTT TTT TTT TTT TTT TTT TTT TTT TTT TTT TTT GTT CAT TTT TTG TTC ATG CCT CGC TGC CGT CGC CA	HS1 and HS2 6bp homology
1X 6bp end pdT	TTT TTT TTT TTT TTT TTT TTT TTT TTT TTT TTT TTT TTT TTT TTG TTC ATG CCT CGCTGC CGT CGC CA	HS1 6bp homology
1X 6bp mid pdT	TTT TTT TTT TTT TTT TTT TTT TTT TTT TTT TTT GTT CAT TTT TTT TTT TTG CCT CGC TGC CGT CGC CA	HS2 6bp homology
2X 7bp pdT	TTT TTT TTT TTT TTT TTT TTT TTT TTT TTA CGT TCA TTT TTT TCG TTC ATG CCT CGC TGC CGT CGC CA	HS1 and HS2 7bp homology
2X 8bp pdT	TTT TTT TTT TTT TTT TTT TTT TTT TTT TTA CGT TCA TTT TTT ACG TTC ATG CCT CGC TGC CGT CGC CA	HS1 and HS2 8bp homology
31+	ATG AGC GCC ACT TTT CAT CAC GTT GTT AGA T	For dsDNA 31bp
31-	ATC TAA CAA CGT GAT GAA AAG TGG CGC TCA T	31+ complement

39+	ATG AAC GTC GCG GGT GAT CTG AAT ATC AAT CTC TAA GCT	For dsDNA 39bp
39-	AGC TTA GAG ATT GAT ATT CAG ATC ACC CGC GAC GTT CAT	39+ complement
45+	GCA TAC ATG AAC GTC GCG GGT GAT CTG AAT ATC AAT CTC TAA GCT	For dsDNA 45bp
45-	AGC TTA GAG ATT GAT ATT CAG ATC ACC CGC GAC GTT CAT GTA TGC	45+ complement
60+	ATC TAA CAA CCT GAT GAA AAG ATG AAC GTC GCG GGT GAT CTG AAT ATC AAT CTC TAA GCT	For dsDNA 60bp
60-	AGC TTA GAG ATT GAT ATT CAG ATC ACC CGC GAC GTT CAT CTT TTC ATC AGG TTG TTA GAT	60+ complement
